# A Self-Managed Online Mindfulness Program in a University-Wide Learning Management System Orientation Site: A Real-World Ecological Validation Study

**DOI:** 10.3389/fpsyg.2022.869765

**Published:** 2022-05-06

**Authors:** Jennifer Chung, Matthew E. Mundy, Stephen McKenzie

**Affiliations:** ^1^School of Psychological Sciences, Monash University, Melbourne, VIC, Australia; ^2^Monash Centre for Professional Development and Monash Online Education, Monash University, Melbourne, VIC, Australia; ^3^School of Psychological Sciences, The University of Melbourne, Melbourne, VIC, Australia

**Keywords:** mindfulness intervention, online study mode, student wellbeing, online intervention, ecological study, real-world implementation, higher education, stress

## Abstract

The efficacy of mindfulness-based interventions in randomised-controlled trials and large experimental studies has been demonstrated in university student populations. Whilst these investigations have provided insight into the nature of the delivery of mindfulness-based practices, there has been little research in the implementation of self-managed online student wellbeing and mindfulness programs at university. This ecological validation study conducted in 2020 evaluated a real-world implementation of a large, university-wide, online mindfulness-based program that was accessible fully online via the tertiary institutions’ Learning Management System (LMS) student orientation site. The total sample included 833 participants from a range of disciplines and faculties at Monash University, Australia. At the end of the study, 236 (28.3%) participants were retained and completed the follow-up survey. Participants had the option to engage with the fully self-managed online mindfulness program for a 12-week semester. The mindfulness practices were pre-recorded, audio-guided sessions, and 10–15 min in length. Baseline and end of semester questionnaires included the 14-item Warwick-Edinburgh Mental Wellbeing Scale, 10-item Perceived Stress Scale and the 18-item Five Facet Mindfulness Questionnaire. Participants who engaged with the mindfulness program over 3 or more weeks showed significant improvements in all three outcome measures, and all participants showed significant improvements in wellbeing at the end of semester. Learning analytics obtained via the LMS revealed that 58.6% (*n* = 489) had not logged into the mindfulness program at all, almost a third (31.0%, *n* = 259) logged into the program materials once or twice, and 10.2% (*n* = 85) of the whole sample engaged with the program actively, having logged in three or more times. The total number of student logins peaked in week 2, reduced between week 2 and week 7 and thereafter activity remained stable until the end of the semester. We hypothesise that the changes in wellbeing, stress and mindfulness at the end of the semester seen in the low engagement participants may partly be explained by the circumstances of COVID-19 restrictions improving. This study has revealed and discusses the complexities of student behaviour and implications for implementing an online mindfulness program in the real- world setting of a university.

## Introduction

The wellbeing of university students has been an important topic of discussion and research over the last 10–15 years, with an exponential increase in recent years (Rickwood et al., 2016). Student wellbeing is a concern because whilst a smaller proportion of students have diagnosed mental health conditions, many others may fall into subclinical levels of stress and anxiety, and more generally, reduced wellbeing (Stallman, 2010; Schofield et al., 2016). Course content (Leslie and Hutchinson, 2018; Arulkadacham et al., 2021) and assessment (McIntyre et al., 2018; Wass et al., 2020) can be associated with distress which is in turn associated with lower academic performance (Stallman, 2010). However, whilst this distress may be high, on-going and impact educational outcomes, research suggests that it does not necessarily impact on dropout rates (Stallman et al., 2019). Furthermore, school-leavers/younger students are also transitioning into adulthood, commencing employment and taking on other responsibilities (Parker et al., 2004). Older or mature age students (those over 21 years of age) are typically juggling other challenges, often including full time employment, carer responsibilities, financial stress and other intangible impacts such as time and guilt concerns as a result of other life situation characteristics (e.g., carer responsibilities) (Stone and O’Shea, 2013).

Finally, a growing subpopulation of students are those that are enrolled fully online and learn remotely. Prior to the onset of forced remote learning due to COVID-19, more students worldwide are opting for either mixed mode or fully online study. It has been estimated that over seven million (in 2020) and half a million (in 2019) students in the United States and Australia, respectively, were enrolled in mixed, distance or online modes (Seaman et al., 2018; Australian Government Department of Education Skills and Employment, 2020; U.S. Department of Education, 2020). The wellbeing of fully online students is under-researched; however given the primary benefit of online study is its flexibility, it often attracts mature age students (Bailey et al., 2015; Johnson, 2015). We know that mature age students are juggling many other responsibilities which make formal university learning challenging. Furthermore, fully online study is likely to result in novel stressors as a result of the unique environment and often intensive study structure. Therefore, it is essential that we address the wellbeing of all university students, including this smaller yet growing subgroup of online students.

### Supporting Student Wellbeing in Universities

To address student wellbeing and assist students with developing and maintaining positive healthy habits, more institutions have started to make wellbeing a priority area, developing formal strategies and frameworks to tackle student mental health (Baik et al., 2016; King’s College London, 2018; Stallman et al., 2019; The University of Sydney, 2020). A common approach is free of cost or part subsidised face-to-face counselling and psychological services. However, the capacity of these services may be limited (Světlák et al., 2021), wait times for non-urgent problems can be lengthy, face-to-face counselling may not be appropriate for everyone (Wong et al., 2018), stigmatisation is still a concern for some students (Martin, 2010) – particularly male students (Rafal et al., 2018), and finally on-campus or face-to-face services are likely to be inaccessible for fully online and remote learning students (Chung and McKenzie, 2020; Minutillo et al., 2020).

To address the limitations of face-to-face counselling services, as well as provide resources as preventative wellbeing measures, some higher education institutions are beginning to offer online wellbeing interventions or programs (Stallman et al., 2019). There is increasing acceptance of online wellbeing resources which may be potentially due to reasons that it can be likened to online education itself including ease of access, flexibility, and scalability. Universities vary, however, in terms of their commitment to student wellbeing, online resources in general and online wellbeing resources. A potential silver lining as a result of COVID-19, is that there has recently been acceleration in the genuine recognition of the value of all of these.

Nevertheless, recent meta-analyses have shown that online wellbeing interventions or programs can be effective and feasible for a range of conditions as well as for improving academic performance (Harrer et al., 2019; Bolinski et al., 2020). There is large variation in the quality, time-involvement and framework of these online wellbeing programs and resources, as well as variation in how they are accessed by students. These resources are not necessarily available to all students either. For instance, in some universities, wellbeing resources (either online or face-to-face) may be provided at a local level to students in individual programs (generally implemented by the coordinators), others at department or faculty level, and lastly in some universities, institution-wide resources may also be available. Finally, as these resources are often part of university strategic goals, evaluation may be primarily scholarship of teaching research and are often not published in peer reviewed academic journals. Therefore, the success of these programs is unclear. A greater understanding of the development and evaluation of these wellbeing resources is fundamental to help ensure evidence-based practice, allowing a greater sharing of resources as well as collaboration between institutions.

### Mindfulness in an Online Context

The value of mindfulness as a wellbeing enhancing practice is becoming more commonly accepted and adopted by the wider population (McKenzie and Hassed, 2012), as well as by institutions aiming to provide wellbeing support (Cavanagh et al., 2013; Světlák et al., 2021). Mindfulness has been described as the process of fully attending to and accepting one’s present moment experiences (for an in-depth review, see Kabat-Zinn, 2003; McKenzie and Hassed, 2012; Creswell, 2017). Mindfulness practice has been successfully incorporated into mindfulness-based interventions (MBIs) which help people develop an openness and receptivity to experience, and to be non-judgement towards the practice itself as well as towards oneself and other people (Antonova et al., 2021).

Cavanagh et al. (2013) explains that the most common mindfulness-based interventions that have been thoroughly evaluated are mindfulness-based stress reduction (MBSR) and mindfulness-based cognitive therapy (MBCT). Both of these variations are based upon eight mindfulness sessions being administered in group therapy settings. Another form of mindfulness-based intervention is low intensity mindfulness-based self-help (MBSH) interventions. Generally these are characterised by reduced practitioner time and resources, being more widely available, and sometimes include books, audio guides, online programs or smartphone applications (“apps”). Cavanagh et al.’s (2014) systematic review and meta-analysis demonstrated positive support for MBSH interventions and concluded that they produced significant benefits in improved mindfulness, depression and anxiety levels compared to controls. Similarly, Spijkerman et al.’s (2016) meta-analytic review of 15 randomised controlled trials revealed support for online MBIs, and also demonstrated that the largest effect was on stress, with a moderate effect size. There is also now increasing research evidence for the efficacy and value of mindfulness apps (e.g., Smiling Mind, 2021; Headspace, 2022) for improving wellbeing (Howells et al., 2016; Flett et al., 2019; Gál et al., 2021; Lahtinen et al., 2021; Orosa-Duarte et al., 2021).

There is significant evidence supporting the benefits of mindfulness for improving anxiety, stress, and wellbeing in both university and/or non-clinical samples (e.g., Kaviani et al., 2011; Demarzo et al., 2017; Cavanagh et al., 2018; Querstret et al., 2018; Chung et al., 2021). Although the mechanisms of mindfulness interventions are complex and also not fully understood yet, it has been established that mindfulness impacts on two main biological pathways (Creswell and Lindsay, 2014; Creswell et al., 2019). Firstly, it enhances the regulatory pathway that increases functional connectivity and activity in stress related regions in the prefrontal cortex. Secondly, it dampens the stress-reactivity pathway (in brain regions such as the amygdala and the anterior cingulate cortex) (Creswell and Lindsay, 2014; Creswell et al., 2019). The primary psychological mechanisms which mindfulness impacts is reactivity to repetitive thoughts, enhancing emotion regulation skills, fostering positive coping strategies (e.g., positive reappraisal) and increased resilience (Teasdale et al., 1995; Epel et al., 2009; Feldman et al., 2010; Hanley et al., 2015; Bamber and Kraenzle Schneider, 2016; Galante et al., 2018; Hwang et al., 2018). Mindfulness has demonstrated decreases in stress and improvements in wellbeing as a result of the biological and psychological pathways that it impacts.

The above listed benefits of mindfulness support its use by universities to provide support for and promote wellbeing in their students (Altinyelken et al., 2020; Světlák et al., 2021). A range of mindfulness-based interventions or programs have been evaluated in randomised controlled trials conducted with university students (for a systematic review, see McConville et al., 2017). For example, de Sousa et al. (2021) evaluated the impact of a brief, group based, daily 30-min recorded audio sessions administered in a 3-day intervention. Dvořáková et al. (2017) utilised the mindfulness program *Learning to BREATHE* which consisted of the administration of eight group sessions (with 20–25 students) over 6 weeks, with each session lasting approximately 80 min. Hall et al. (2018) assessed an intervention that consisted of two face-to-face guided sessions and weekly self-guided practice for 7 weeks. Finally, Orosa-Duarte et al. (2021) evaluated a smartphone app that was made up of eight stages, each with short videos and audio sessions, providing a total of more than 200 min of session time.

Yet there is little to no evidence of how well mindfulness-based programs are implemented as a student resource in a wider university setting, when not directly targeted to a selected group of students undergoing a research trial. In other words, would students actually engage and if so, how would they engage with a mindfulness-based program that is ‘freely available’, similar to other university resources (e.g., library support services)? There is therefore a need to explore the implementation of mindfulness programs for university students in a naturally occurring, ecologically valid, real-world setting. Also, given the growing prevalence of online education and the special wellbeing needs of online students there is a particularly strong need to explore online mindfulness programs for university students.

### The Current Study

The current study builds on the findings from Chung et al. (2021), where the quasi-experimental evaluation supported a brief, online, mindfulness-based intervention for the wellbeing, perceived stress and mindful attention of on-campus and fully online students. The current study evaluated a large institution-wide implementation of a mindfulness-based program that is accessible fully online via a tertiary institutions’ Learning Management System (LMS), and housed within the university-wide student orientation site. For context, this study was conducted at a public and Australia’s largest university with over 85,000 total enrolled students (64% are undergraduates) and over 15,000 who are enrolled off-campus or multi-modal (Monash University, 2022).

#### COVID-19 Context

This research was conducted in August, 2020. COVID-19 has had large implications on society, and resulted in university students expressing feelings of lack of motivation, anxiety, stress, and isolation, as well as behaviour changes such as social distancing and socialising less, as well as education and enrolment changes (Browning et al., 2021). In Victoria (the Australian state of the university where this research study was conducted), state-wide stay-at-home restrictions (unless deemed essential) were imposed between early July and late October 2020. University students were required to study fully online, remotely, without any attendance on-campus.

During COVID-19, it has been reported that dispositional mindfulness was associated with lower levels of pandemic distress (Conversano et al., 2020) and fear of COVID-19 itself (Belen, 2021). Furthermore, the evaluation of mindfulness-based online programs during the pandemic have supported their value in reducing anxiety, stress and increased self-compassion (González-García et al., 2021; Simonsson et al., 2021; Sun et al., 2021).

The experiences during COVID-19 due to a worldwide pandemic was at a population level and is extremely novel and rare. Understanding and evaluating the uptake of wellbeing resources during this extreme event can potentially provide insight into student behaviours at other high stress periods. For example, students at university might be in a heightened state of stress whilst enrolled in a semester, but potentially may experience a further heightening just prior to an assignment deadline, and again even further when preparing for end of semester exams. These stressors could be referred to as *standard academic stressors*. Other stressors that university students are likely to experience are *standard personal stressors* such as personal, health or relationships problems. This research may provide useful insight into student behaviour during *standard academic* and *standard personal* high stress times, as well as the possible interaction of *both* of these occurrences.

#### Rationale, Research Aims and Questions

In summary, there is strong evidence to for the use of online mindfulness-based programs to support student wellbeing. However, as Kelly (2012) points out, implementation science addresses the “problems [that] have arisen in the transfer of these [programs] to real-world contexts that lack experimental control” (p. 4). To the authors’ knowledge, there is little or no available evidence of real-world, large-scale, university-wide implementations of such wellbeing programs. Additionally, there is a lack of insight into program engagement via examination of learning analytics. Finally, exploring the uptake and potential impact of a mindfulness based online program during COVID-19 (unprecedented time period at a population level) is valuable in itself, as it may improve understanding of students’ behaviours and intentions in other stressful periods of university life.

The primary aim of this ecological validation and real-world study was to understand patterns of student engagement with the Monash Online Mindfulness Program available via the university’s LMS university-wide orientation site. A secondary aim was to understand the impact of engagement patterns with the Monash Online Mindfulness Program on student wellbeing, stress and mindful attention. Across both of these research aims, we acknowledge the specific context of COVID-19 restrictions and lockdowns and consider how this may impact student engagement.

The following questions guided our research:

**RQ1.** Is the university-wide LMS-based Monash Online Mindfulness Program associated with changes in student wellbeing, stress and mindfulness, and is its impact associated with engagement levels?

**RQ2.** How do learning analytics help us understand the nature of students’ engagement with the university-wide LMS-based Monash Online Mindfulness Program?

**RQ3.** Based on the Theory of Planned Behaviour (Ajzen, 1991), what are barriers and motivations for engaging in the university-wide LMS-based Monash Online Mindfulness Program and are these barriers and motivations associated with participants’ engagement levels?

## Materials and Methods

### Participants

Participants were recruited via university-wide student orientation LMS Moodle sites (known as ‘Monash Essentials’) in Semester 2, August 2020. The Monash Essentials sites were only available to newly commencing Monash students in Semester 1 and Semester 2, 2020, and there were separate sites for students in the two semesters. This Monash Essentials site contained orientation resources and information, as well as the Monash Online Mindfulness Program. All students were invited via an initial and reminder invite in a dedicated discussion forum post (within Monash Essentials) about the study. Additionally, students that were new in Semester 2 (enrolled in the Semester 2 site) were also sent a central university email regarding Monash Essentials. This email also contained one statement inviting students to participate in this mindfulness study. Students who were enrolled in the online study mode were invited via one email invitation sent by the student support team.

Students invited to participate in the study were from a range of disciplines, in the first year of their undergraduate or graduate course, enrolled in any Monash University campus (locations in Australia, Malaysia, China, India, and Italy) and in either on-campus or online study modes.

In total, 19,700 and 1,500 students were invited/enrolled in the Semester 1 and Semester 2 Moodle sites, respectively. A total of 833 participants (Sem 1: *n* = 494; Sem 2: *n* = 339) provided consent to participate in the study and completed the baseline survey. Therefore the response rate for Semester 1 students was 2.51%, for Semester 2 students it was 22.6%, and finally the overall response rate was 3.93%. During the study period, 236 (28.3%) participants were retained and completed the follow-up/end of semester survey. The demographics and characteristics of the sample is shown in Table 1.

**TABLE 1:** Participant characteristics as a percentage of the sample.

	Total (*N* = 833)
**Age**	
Mean (SD)	24.51 (7.61)
Range	18–62 years
**Gender*^a^***	
Female	605 (72.6%)
Male	222 (26.7%)
Non-binary	5 (0.6%)
**Study mode**	
On-campus	708 (85.0%)
Online	125 (15.0%)
**Course level**	
Undergraduate	412 (49.5%)
Single degree	304 (73.8%)
Double degree	108 (26.2%)
Postgraduate	421 (50.5%)
Coursework	357 (84.8%)
Research masters	46 (10.9%)
Doctorate/Ph.D.	18 (4.3%)
**Enrolment type**	
Domestic	445 (53.4%)
International	388 (46.6%)
**Enrolment status**	
Full-time	757 (90.9%)
Part-time	76 (9.1%)
**Faculty**	
Art, Design and Architecture	26 (3.1%)
Arts	85 (10.2%)
Business and Economics	167 (20%)
Education	115 (13.8%)
Engineering	40 (4.8%)
IT	58 (7.0%)
Law	23 (2.8%)
Medicine, Nursing and Health Sciences (including psychology)	197 (23.6%)
Pharmacy and Pharmaceutical Sciences	36 (4.3%)
Science	82 (9.8%)
Other	4 (0.5%)
**Current location during COVID-19*^a^***	
University’s home city, in Australia	602 (72.3%)
At home, not in Australia	97 (11.6%)
At home in Australia, but not in University’s home city	84 (10.1%)
University Campus	34 (4.1%)
**Experience**	
Meditation, mindfulness, yoga or other contemplative activity (Yes)	574 (68.9%)
Meditation (Yes)	447 (77.9%)
Mindfulness (Yes)	402 (70.0%)
Yoga (Yes)	416 (72.5%)
Other (Yes)	203 (35.4%)
**Mindfulness minutes per week**	
I inconsistently practice mindfulness	186 (46.3%)
I do not normally practice mindfulness	89 (22.1%)
Up to 30 min per week	46 (11.4%)
Between 30 min to 1.5 h per week	58 (14.4%)
More than 1.5 h per week	23 (5.7%)

*Percentages are based on valid percent.*

*^a^Respondents also had the opportunity to indicate ‘Other’, or ‘Prefer not to say’.*

Participation was voluntary and inclusion criteria included students being over the age of 18. Participation was not anonymous. Participants were given the opportunity to enter a draw to win 1 of 15 $50 AUD gift vouchers.

#### COVID-19 Affected Factors

Participants’ residential location (in relation to the primary city of the University’s campus) during the intervention period/COVID-19 is presented in Table 1. The majority of the sample (72.3%) were residing in Melbourne, Australia, and were experiencing the second major lockdown which spanned approximately 4 months. Students who were enrolled on-campus were studying remotely for all or most of the semester and study period. Students enrolled in the online study mode included those who are not required to come onto campus as part of their program and study entirely remotely (regardless of COVID-19).

### Procedure

Monash University Human Research Ethics Committee approval was obtained prior to the commencement of the study. At the start of the semester, students were invited to voluntarily participate in the study via the ‘Monash Essentials’ LMS Orientation site. Prospective participants reviewed the explanatory statement and provided implied consent by commencing the survey. Participants completed the baseline/start of semester survey via the online survey platform, Qualtrics. Participants were then instructed to self-enrol into the Mindfulness program, a section created for this research study, within the Monash Essentials LMS site.

Participants were encouraged to engage with the mindfulness program throughout the semester, however this was not required for participation in the study. At the end of the 12-week semester (14 weeks including 2 weeks of mid-semester break) all participants were asked to complete the end of semester/follow-up survey.

### Materials and Measures

#### The Monash Online Mindfulness Program

The Monash Online Mindfulness Program was developed based on established evidence-based mindfulness-based interventions including Chung et al. (2021). This program can be categorised as a MBSH intervention due to its online nature and relatively little practitioner involvement (Cavanagh et al., 2014). It was appropriate for a new MBSH intervention to be created that was specific and tailored to a University setting given its unique nature, as well as to the LMS orientation site in which this program was housed within. It was not appropriate for a structured 8-week MBSR to be implemented in this context, as a brief, introductory level and low perceived burden was deemed essential for encouraging students to participate in this program. The Monash Online Mindfulness Program was delivered using the university’s LMS, Moodle, and accessed via the ‘Monash Essentials’ Orientation site for new students.

The Program consisted of three main sections: *Introduction*, *Program 1: Weekly Mindfulness*, and *Program 2: Daily Mindfulness*. The program content opened into a new browser window when it was accessed and was seamlessly integrated into the Monash Essentials LMS site. The Monash Online Mindfulness Program content was created using Articulate Rise 360, an eLearning authoring tool allowing a modern, dynamic course design. Articulate Rise 360 courses were imported as Sharable Content Object Reference Model (SCORM) packages into the LMS site which allowed engagement analytics to be collected. The three sections could be accessed at any time, and participants could navigate the program at their own pace.

The *Introduction section* in the Monash Online Mindfulness Program provided an overview of mindfulness, and briefly explained the benefits of mindfulness and its relationship to physical, mental and emotional health. It introduced participants to the mindfulness presenters (including photo, credentials and a brief biography) and described two variations of the guided mindfulness program that were offered.

The mindfulness program was fully asynchronous and consisted of two variations. The mindfulness practices themselves were consistent across the two programs, although with varying amounts of mindfulness per week. The *Weekly Mindfulness* variation resembled a ‘low intensity’ program and consisted of one guided mindfulness activity per week. This program was suggested for individuals who wanted an introduction to mindfulness. The *Daily Mindfulness* variation resembled a ‘high intensity’ program and consisted of five guided mindfulness activities per week. This program was suited to individuals who wanted an introduction to mindfulness and/or had an intention of dedicating more time to practice.

The mindfulness practices consisted of four pre-recorded audio-guided mindfulness exercises, each between 10–15 min in length. The *body scan* (variation 1) and *coming to your senses* practices were developed and delivered by a male PhD-level psychological researcher (McKenzie, S.) with over three decades of experience engaging in and teaching mindfulness-based practices to university and general populations. The *body scan* (variation 2) and *mindful eating* practices were developed and delivered by a female registered psychologist specialising in mindfulness, resilience and body image. The *body scan* activities focussed on the present moment and awareness of internal experiences in the body. *Coming to your senses* and *mindful eating* focussed on awareness of the senses and being fully aware of each of the senses used in the experience of eating something, or drinking something, respectively. A brief description of each practice and its benefits was provided in written text accompanying the audio, as well as at the start of each session.

#### Survey

Participants completed two self-report surveys. The baseline questionnaire consisted of (1) basic demographic information (name, student ID, age, and gender), (2) student characteristics (years enrolled in current degree, faculty, course level, enrolment type, enrolment status, study mode and campus location), (3) previous experience with meditation, mindfulness, yoga, and other contemplative activities, (4) three validated scales measuring wellbeing, perceived stress, and mindfulness, described below.

The end of semester questionnaire consisted of (1) the same three validated measures, (2) impact of COVID-19, (3) barriers and motives of participating in mindfulness, (4) feedback on the Monash Online Mindfulness Program. Items in components 2–4 were generated by the researchers for this research study and participants were invited to respond via selecting choice options (i.e., not free text). The impact of COVID-19 items were developed based on the general literature that had been published on challenges students faced in light of COVID-19. Six items (three each) measured the barriers and motives of participating in mindfulness interventions, and were developed based on The Theory of Planned Behaviour literature (Ajzen, 1991; Armitage and Conner, 2001). These items were rated on a five-point Likert scale (*1* = *strongly disagree*, *5* = *strongly agree*).

##### Warwick-Edinburgh Mental Wellbeing Scale

The Warwick-Edinburgh Mental Well-being Scale (WEMWBS) is a 14-item scale measuring well-being over the past 2 weeks (Tennant et al., 2007). Items are measured on a five-point Likert scale (*1* = *none of the time* to *5* = *all of the time*). Items were summed producing a total score between 14 – 70. Higher total WEMWBS scores indicate increased wellbeing. Cronbach’s alpha has been reported as 0.89–0.91 in university student and population samples, as well as high test–retest reliability (α = 0.82; Tennant et al., 2007). Cronbach’s alpha in the current sample was 0.90, and is considered high and acceptable. The WEMWBS has been used in evaluations of MBIs such as in Galante et al. (2018),Clarke and Draper (2020), Chung et al. (2021), and Trottier et al. (2021).

##### Perceived Stress Scale

The Perceived Stress Scale (PSS) is a commonly used 10-item scale measuring the perception of stress (Cohen et al., 1983). Participants rated items on a five-point Likert scale (*0* = *never* to *4* = *very often*), over the past month. Four items are reverse scored, all items are summed to produce a total PSS score between 0 and 40. PSS scores ranging from 0 to 13, 14 to 26, and 27 to 40 indicate low, moderate and high perceived stress, respectively. Cronbach’s alpha indicates high reliability with alpha coefficients of 0.84–0.86, and test–retest reliability correlation of 0.85 in college students (Cohen et al., 1983). In the current sample, Cronbach’s alpha is also high at 0.86. The PSS has been used in many evaluations of MBIs, for example Cavanagh et al. (2013, 2018) and Querstret et al. (2018).

##### Five Facet Mindfulness Questionnaire-18

The Five Facet Mindfulness Questionnaire 18-items (Medvedev et al., 2018) is a modified version of the full 39-item measure (Baer et al., 2006). Participants rated items on a 5-point liker scale (*1* = *never or very rarely true* to *5* = *very often or always true*). The FFMQ is a global measure of the mindfulness construct with five subscales: Act with Awareness, Describe, Non-judge, Non-react, and Observe. Nine items are reverse scored and all items are summed producing a total score, however the individual subscales are not reported in this study. Cronbach’s alpha in the current sample at 0.75 is considered acceptable for research purposes. The FFMQ has been used to assess MBIs such as in De Vibe et al. (2013), Shore et al. (2018), and Světlák et al. (2021).

#### Learning Analytics

Student login and activity data were collected from the Moodle LMS sites that hosted the Monash Online Mindfulness Program. Each time an individual entered the program, a unique login was recorded. An export file of log data provided the student ID, name, access dates, and login duration, for each unique login.

In total, LMS log files were available for each of the 12 weeks of the intervention program and 2 weeks of the mid-semester break. The LMS log files were collated into a single Excel file. To measure the pattern of logins across the semester, the weeks logged in, and the total number of weeks logged in were collated. Whilst duration of login was also recorded, this data merely consists of the time across which the LMS program was open on the device, and does not include other more relevant activity (e.g., clicks, scrolling, play/pause on audio files). Therefore, duration of login may not accurately represent engagement with the Monash Online Mindfulness Program and thus such data will not be reported.

### Design

The study followed a quasi-experimental, single group, pre-test – post-test design. To evaluate the ecological validity of the Monash Online Mindfulness Program, and to evaluate the natural engagement of wellbeing enhancing resources in a higher educational context, a single group design, without a control group, was used. Participants were asked to provide their Student ID so that survey responses could be matched to student log data collected from the Moodle LMS. As such, participation in this study was not anonymous.

All three research questions are addressed in part by the learning analytics collected from the Moodle LMS sites. RQ1 is also addressed by the WEMWBS, PSS, and FFMQ scales. RQ2 is addressed only by the learning analytics. RQ3 is addressed by the six items measuring the barriers and motives of participating in mindfulness.

### Data Handling and Statistical Analysis

The survey data and student log data files were downloaded from Qualtrics and the Moodle LMS site, respectively. Survey data was imported into SPSS Version 27, and appropriate data cleaning was conducted. The Moodle LMS log file report was imported into SPSS and merged with survey data based on student ID that was available in both files.

An engagement variable was computed based on *Total number of program weeks accessed*, obtained from Moodle LMS log data. A pragmatic rationale was used to determine participants’ engagement levels. We deemed participants who had logged in once or twice as demonstrating superficial engagement, and therefore their logins were of a ‘trial’ nature. Participants who had logged in three or more times were deemed as logging in to engage with the materials, and therefore demonstrated ‘active’ engagement. The engagement variable had three levels: *No engagement* (zero logins), *Trial engagement* (1–2 logins), and *Active engagement* (3 or more logins).

## Results

The baseline, follow-up and corresponding change scores of the WEMWBS, PSS, and FFMQ of the total sample are presented in Table 2.

**TABLE 2:** Baseline, follow-up and change scores in wellbeing, stress and mindfulness.

	*n*	Baseline	*n*	Follow-up	Change
WEMWBS	833	45.38 (8.30)	236	48.10 (8.44)	2.60 (8.37)
PSS	833	20.72 (5.83)	230	18.30 (5.90)	−1.51 (5.56)
FFMQ	833	54.45 (8.70)	229	57.87 (8.71)	2.76 (8.39)

*Data are Mean (SD). Change scores are Follow-up scores – Baseline scores. WEMWBS, Warwick, Edinburgh Mental Wellbeing Scale; PSS, Perceived Stress Scale; FFMQ, Five Facet Mindfulness Questionnaire.*

### Change in Wellbeing, Stress and Mindfulness

#### Within Engagement Type

In Table 3, the mean differences of change in the outcome measures for each of the three groups of engagement type are presented. Paired samples *t*-tests were conducted for the outcome measures of WEMWBS, PSS and FFMQ, for each of the groups of participants across the engagement types (*No engagement*, *Trial engagement*, *Active engagement*).

**TABLE 3:** Mean differences of change in wellbeing, stress and mindfulness in groups of engagement type.

No engagement users (zero attempts)											
				Paired samples *t*-test							
						95% CI					
	*n*	Baseline	Follow-up	*Mean difference*	*Std err mean*	Lower	Upper	*t*	*df*	*p*	Cohen’s *d*
WEMWBS	103	45.83 (8.87)	47.79 (8.75)	1.95 (8.53)	0.84	0.28	3.62	2.32	102	**0.022**	0.23
PSS	99	19.88 (5.46)	19.28 (5.68)	−0.60 (5.56)	0.56	–1.7	0.51	–1.07	98	0.289	0.11
FFMQ	99	54.69 (8.70)	57.06 (8.49)	2.37 (7.25)	0.73	0.93	3.82	3.26	98	**0.002**	0.33

**Trial engagement users (1–2 attempts)**											

				**Paired samples *t*-test**							
						**95% CI**					
	***n***	**Baseline**	**Follow-up**	***Mean difference***	***Std err mean***	**Lower**	**Upper**	***t***	***df***	***p***	**Cohen’s *d***

WEMWBS	76	44.49 (8.59)	47.25 (8.41)	2.76 (7.66)	0.88	1.01	4.51	3.15	75	**0.002**	0.36
PSS	74	20.36 (6.29)	18.23 (6.18)	-2.14 (5.22)	0.61	–3.34	-0.93	–3.52	73	**<0.001**	0.41
FFMQ	73	55.52 (9.14)	57.34 (8.88)	1.82 (8.62)	1.01	–0.19	3.83	1.81	72	0.075	0.21

**Active engagement users (3 or more attempts)**											

				**Paired samples *t*-test**							
						**95% CI**					
	***n***	**Baseline**	**Follow-up**	***Mean difference***	***Std err mean***	**Lower**	**Upper**	***t***	***df***	***p***	**Cohen’s *d***

WEMWBS	57	46.26 (8.01)	49.81 (7.79)	3.54 (8.99)	1.19	1.16	5.93	2.98	56	**0.004**	0.39
PSS	57	18.98 (5.20)	16.68 (5.63)	-2.30 (5.87)	0.78	–3.86	-0.74	–2.96	56	**0.005**	0.39
FFMQ	57	55.35 (8.97)	59.96 (8.69)	4.61 (9.71)	1.29	2.04	7.19	3.59	56	**<0.001**	0.48

*Data are Mean (SD). Bold = significant p < 0.05. WEMWBS, Warwick, Edinburgh Mental Wellbeing Scale; PSS, Perceived Stress Scale; FFMQ, Five Facet Mindfulness Questionnaire; CI, confidence interval.*

Participants in the *No engagement* group did not differ on PSS between follow-up and baseline. However, significant differences were found for the other two outcome measures of WEMWBS and FFMQ, where follow-up scores were 1.95 and 2.37 points higher than baseline scores, and corresponded to small effect sizes of 0.23 and 0.33, respectively. The participants in the *Trial engagement* group showed a significant mean increase of 2.76 and decrease of 2.14 scores in WEMWBS and PSS, respectively. Cohen’s *d* effect sizes of 0.36 for WEMWBS and 0.41 for PSS were obtained, which is described as small to medium. The paired samples *t*-test for the *Trial engagement* group was not significant for FFMQ. Finally, the participants in the *Active engagement* group showed significant differences in all three outcome measures of WEMWBS, PSS, and FFMQ from baseline to follow-up. On average, participants follow-up WEMWBS scores were 3.54 points higher, PSS scores were 2.30 points lower, and FFMQ scores were 4.61 points higher than at baseline. These effect sizes were all small to medium at 0.39, 0.39, and 0.48 for WEMWBS, PSS, and FFMQ, respectively.

#### Between Engagement Types

After reviewing the Kolmogorov-Smirnov values, histograms and P-P plots, it was determined that the distributions of change scores for WEMWBS, PSS, and FFMQ in each of the engagement group types violated normality. Therefore, a non-parametric Kruskal–Wallis ANOVA analysis was conducted on the change scores for WEMWBS, PSS, and FFMQ between the three engagement types. Although the mean ranks in scores between follow-up and baseline appear to increase in trend from the least engaged to the most engaged, no statistically significant differences were found in any of the change scores of outcome measures between the three groups, of *No engagement*, *Trial engagement*, and *Active engagement* (see Supplementary Material 1).

### The Monash Online Mindfulness Program

#### Learning Management System Moodle Analytics

Learning Management System Moodle analytics revealed that 37.9% (*n* = 316) of the sample logged in to access the *Introduction* section of the program. The 41.3% (*n* = 344) of the total sample accessed the *Weekly* or *Daily* version Mindfulness programs at least once (*M* = 2.00 weeks, *SD* = 2.21) across the semester/intervention period. A total of 344 (41.3%) participants accessed the *Weekly* program (*M* = 1.47 weeks, *SD* = 1.16), and 237 (28.5%) accessed the *Daily* program (*M* = 2.23 weeks, *SD* = 2.18). Finally, 27.6% (*n* = 230) of the sample ticked/marked the sessions as ‘*complete*’ at least once in the intervention period (*M* = 1.52, *SD* = 1.29). Overall, there were 2,142 unique logins into the Mindfulness program.

**FIGURE 1:**
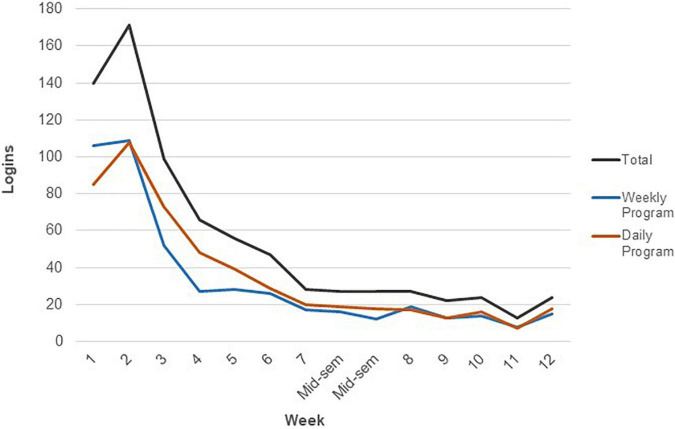
The pattern of mindfulness program login across the semester. The data represents the total frequency of logins in the sample for each week of the semester. Mid-sem, Mid-semester break.

The pattern of logins across the weeks in the semester is presented in Figure 1. As depicted in Figure 1, the number of program accesses is highest in the first few weeks of the semester (week 1–3), peaks in week 2, declines in week 3–4 and week 4–7, approximately steady use thereafter until a small reduction in access in week 11, and finally a return to increased access in week 12.

#### Program Feedback

At follow-up, 153 participants (66.8%) self-reported accessing the mindfulness activities during the semester. Table 4 presents the feedback items as responded to by participants who self-reported engaging with the mindfulness activities.

**TABLE 4:** Mindfulness program feedback.

	Total (*N* = 153)
**Difficulties completing activities**	
Technical or internet difficulties (e.g., recording stop/starting/disrupted)	22 (14.4%)
Did not understand or could not follow what to do during the activity	16 (10.5%)
Could not find or navigate through the activities/sessions	11 (7.2%)
Audio files are too loud/not loud enough/inaudible	10 (6.5%)
Other	20 (13.1%)
**Where did you normally complete the mindfulness activities**	
At home in a private/quite spot	144 (94.1%)
At home in a disruptive/busy spot	2 (1.3%)
Outdoors (e.g., backyard, garden, park)	6 (3.9%)
Other	1 (0.7%)
**Voice preference**	
Male voice	26 (17.0%)
Female voice	67 (43.8%)
No preference	30 (39.2%)

*Percentages are based on valid percent.*

### Characteristics of the Sample

#### Baseline Comparisons

The descriptive statistics of baseline scores of WEMWBS, PSS, and FFMQ for each group of engagement types are shown in Table 5. The one-way between groups ANOVA for PSS at baseline was significant, indicating that baseline PSS scores were significantly different between the engagement type groups, η^2^ = 0.01 representing a small effect size. Bonferroni *post hoc* analyses revealed that the *No engagement* group had significantly higher baseline PSS scores than the *Trial engagement* group, *p* = 0.022, and this effect size was small, *d* = 0.19. The *post hoc* analyses between the remaining pairs of groups were not significant. No mean group differences were found for WEMWBS and FFMW at baseline.

**TABLE 5:** Analysis of variance between engagement type groups for baseline wellbeing, stress and mindfulness.

				ANOVA					

	No engagement (*n* = 489)	Trial engagement (*n* = 259)	Active engagement (*n* = 85)	*F*(2,830)	*p*
Baseline WEMWBS	45.17 (8.61)	45.89 (7.78)	44.99 (8.02)	0.744	0.476
Baseline PSS	21.22 (5.85)	20.02 (5.88)	20.04 (5.30)	4.26	**0.014**
Baseline FFMQ	54.30 (8.69)	55.11 (8.46)	53.32 (9.40)	1.541	0.215

*Data are Mean (SD). Bold = significant p < 0.05. WEMWBS, Warwick, Edinburgh Mental Wellbeing Scale; PSS, Perceived Stress Scale; FFMQ, Five Facet Mindfulness Questionnaire.*

#### Barriers and Motives

The items describing three barriers to and three motives for engaging in mindfulness are presented in Table 6. Of the total follow-up sample that responded to these items, 98 participants were *No engagement* users, 73 participants were *Trial engagement* users and 57 participants were *Active engagement* users. Table 6 presents the means, standard deviations and the percentage of the sample that selected agree and strongly agree for each item. A one-way between groups ANOVA was conducted for each item and there were no significant differences found between the three engagement type groups across any of the six barriers and motives items.

**TABLE 6:** Analysis of variance between engagement type groups on barriers and motives.

						ANOVA	

	Total sample (*N* = 228)		No engagement (*n* = 98)	Trial engagement (*n* = 73)	Active engagement (*n* = 57)	*F*(2,225)	*p*
**Barriers**							
Remembering or creating a routine	192 (84.2%)	4.24 (0.91)	4.22 (0.79)	4.15 (1.05)	4.37 (0.92)	0.925	0.398
Finding more time in my schedule	167 (73.2%)	3.94 (1.04)	4.02 (1.00)	3.99 (1.03)	3.74 (1.11)	1.449	0.237
Lack of easily accessing the mindfulness activities on other devices (e.g., phone, tablet) or locations (e.g., on the train)	157 (68.9%)	3.92 (1.09)	3.80 (1.04)	4.04 (1.03)	3.96 (1.24)	1.127	0.326
**Motives**							
Seeing that I am benefiting from mindfulness (e.g., general well-being and stress)	197 (86.4%)	4.33 (0.81)	4.32 (0.78)	4.26 (0.85)	4.46 (0.80)	0.975	0.379
Receiving reminders about the mindfulness program (e.g., alerts, notifications)	146 (64.0%)	3.70 (1.12)	3.70 (1.07)	3.62 (1.19)	3.81 (1.13)	0.463	0.630
If my friends and family were also interested in mindfulness	143 (62.7%)	3.75 (1.07)	3.80 (1.12)	3.71 (1.14)	3.74 (1.09)	0.138	0.871

*In column 1, frequencies of strongly agree and agree are displayed with valid percent in parentheses. In columns 2, 3 and 4, data are mean (SD).*

#### Dropout Analysis

The independent samples *t*-tests conducted between participants who completed the study (both baseline and follow-up surveys) and those that withdrew (completed only the baseline survey) was statistically significant; the *dropout* group reported higher PSS scores at baseline than the *did not dropout* group at baseline (Supplementary Material 2). This effect size was small, *d* = 0.22. The remaining *t*-tests between dropout participants and did not dropout participants was non-significant for WEMWBS and FFMQ and do not indicate that dropout was associated with participants’ baseline scores.

#### COVID-19 Impacts at Follow-Up

The frequency of self-reported COVID-19 related impacts in regards to university study and other impacts are provided in Table 7. More than half the participants reported decreased wellbeing, increased stress and isolation, and approximately one in three experienced financial and income difficulties, and altered their original study plan.

**TABLE 7:** COVID-19 impacts at follow-up.

	Total sample (*N* = 229)
**University/study impact**	
Altered study plan	67 (29.3%)
Candidature length extended	27 (11.8%)
Course/degree has changed	24 (10.5%)
Overload	17 (7.4%)
Underload	14 (6.1%)
Changed major/discipline/degree	14 (6.1%)
Significantly changed direction/plan (research study only)	13 (5.7%)
Other	55 (24.0%)
**Other impacts**	
Experienced isolation (e.g., emotional, physical, psychological)	137 (59.8%)
Decreased wellbeing/increased stress or anxiety	120 (52.4%)
Additional financial burden	89 (38.9%)
Loss or reduction in paid income	73 (31.9%)
Additional household responsibilities	64 (27.9%)
Change in living situation	64 (27.9%)
Additional caring responsibilities	39 (17.0%).
COVID-19 illness or death of family member, friend, or associate	27 (11.8%)
Other	19 (8.3%)

*Percentages are based on valid percent.*

## Discussion

This study is the first to the authors’ knowledge to directly explore the ecological validity of a self-managed, fully asynchronous, student online mindfulness-based program offered within an online university-wide LMS-based orientation site. This research was conducted in 2020 during COVID-19 lockdown and restrictions, and provides insight into student behaviours and engagement during other standard academic (e.g., assignment and exam periods) and standard non-academic (e.g., personal, relationship, health problems) stressful periods during university life.

Whilst there is very strong research support for the effectiveness of mindfulness across a range of settings and impacts, including for university students, the authors cannot find studies comparable to the current that provide a real-world evaluation of a large-scale, university-wide online mindfulness wellbeing intervention or program. As such, we will not be drawing heavily upon similar research studies in the discussion of our findings. Instead, we argue that this research provides a valuable ‘next-step’ in mindfulness intervention for university students’ research. This includes a call for our collective awareness to expand from the general effectiveness of mindfulness programs including as measured by experimental implementations to explorations of the full reality of its real-world implementation.

### The Monash Online Mindfulness Program

#### RQ1. Student Engagement and Student Wellbeing, Stress and Mindfulness

Participants were categorised as having *No engagement* (zero logins), *Trial engagement* (1–2 logins), and *Active engagement* (3 or more logins). In the total sample, we found that 58.7% (*n* = 489) of participants had not engaged with or logged into the mindfulness program at all during the semester, 41.3% (*n* = 344) logged in at least once, almost a third (31.0%, *n* = 259) logged in once or twice during the semester (deeming this type of engagement as superficial), finally 10.2% (*n* = 85) engaged with the program actively, having logged in three or more times.

In each of the three types of mindfulness program engagement (*No engagement, Trial engagement, Active engagement*), a positive and significant improvement in wellbeing levels as measured by WEMWBS was evident at the end of the semester compared to the start of the semester, with small to medium effect sizes. This finding is not consistent with our pilot study (conducted pre-COVID-19) (Chung et al., 2021). Whilst a number of reasons could explain these findings, we provide two possible explanations. Firstly, it is possible that the increase in student wellbeing across the sample is due to students acclimating to online learning throughout the semester. At the start of semester students were likely to experience uncertainty around online learning, lockdown, and disappointment; however, by the end of the semester students may have adjusted their expectations and settled into the new life routine including online learning. A second and related reason could be the removal of stay-at-home restrictions that coincided with the end of semester and follow-up survey.

At the start of the semester (baseline), perceived stress levels (as measured by the PSS; Cohen et al., 1983) of participants’ who did not engage with the program (*No engagement*) were significantly higher, indicating greater stress, than participants who attempted and logged into the program once or twice (*Trial engagement*) during the semester. Furthermore, perceived stress of participants who were characterised as either *Trial* or *Active engagement* with the mindfulness program also significantly improved from end of semester to start of semester by 2.30 and 2.14 points, respectively. This is in comparison to students who had never participated in the mindfulness program (*No engagement*), whose PSS scores did not statistically differ over the semester. It is important to note that the PSS scores for each of the three groups, as well as of the overall sample, remained ‘moderate’ at baseline and at follow-up (Cohen et al., 1983).

To summarise, this means that students who chose not to view or engage with the wellbeing program were significantly more stressed at the start of semester, and those that participated (to varying degrees) were not only already less stressed at the start of the semester, but also their stress levels improved by the end of the semester as well. Although the nearing medium effect sizes for the *Trial* and *Active engagement* groups indicate some, but not large practical implications and significance. Nevertheless, the significant reduction in perceived stress experienced by the *Trial* and A*ctive engagement* groups is consistent with a trial conducted during COVID-19 where participants anxiety decreased after a mindfulness-based intervention (Simonsson et al., 2021; Sun et al., 2021). Greater trait mindfulness (how an individual expresses their everyday mindful attitudes and behaviours) is associated with increased stress resilience and increased wellbeing through facilitating greater psychological flexibility (Weinstein et al., 2009; Pidgeon and Keye, 2014; Bajaj and Pande, 2016). This psychological mechanism potentially explains the findings of this study where participants who engaged with mindfulness showed greater wellbeing and decreased stress (at the end of the semester) due to increased adaptability following the COVID-19 general and educational impacts, as well as general university stresses.

Mindfulness levels as measured by the FFMQ significantly increased in the group of participants who had engaged with the mindfulness program three or more times (*Active* users) corresponding to a medium effect size, as did the *No engagement* group, albeit a small effect size. The FFMQ scores of the *Trial engagement* group did not statistically change over the course of the semester. Whilst perhaps it could be assumed that there would be a linear relationship between greater engagement with the mindfulness program associated with greater mindfulness (and other outcome measures), this real-world implementation does not support this assumption. Are there therefore shared similarities between the *No engagement* group and the *Active engagement* group? Are individuals who trial and make an attempt to engage with mindfulness potentially more negative either before or after their exposure than are the individuals in the other groups? Could the results of participants in this group be influenced by their seemingly making a conscious decision to not continue with the mindfulness program? These possibilities cannot be fully understood based on the findings of this study; however, the possible mechanisms behind this interesting finding is worth exploring in future studies.

Finally, we explored the size of students’ change in wellbeing, stress and mindfulness across the semester, *between* the three types of program engagement. The mean rank differences of the three engagement groups *appear* to be statistically different, as larger mean rank scores of positive changes (higher scores for wellbeing, lower scores for stress, and higher scores for mindfulness) were seen as engagement increases. However, these statistical differences were not significant and change in outcome measures did not differ between the three engagement groups. In other words, whilst improvement in wellbeing was seen across all three groups, one group did not significantly improve more or less than another group. Again, whilst a positive linear relationship may have been expected, our real-world implementation of a self-managed mindfulness program did not reflect this. A more nuanced understanding of this relationship is needed to explain this complex finding. This study was the first to the authors’ knowledge to examine students’ *actual* engagement with a self-managed online mindfulness-based program and subsequently naturally occurring levels of engagement. This research is the first step in exploring the real-world result of a large-scale, university-wide online mindfulness wellbeing intervention. This naturalistic study has therefore resulted in complex findings that cannot necessarily be interpreted in the same way as an experimental study.

#### RQ2. What Is the Nature and Pattern of Student Engagement With the Monash Online Mindfulness Program?

Learning Management System Moodle analytics provided insight into when and how often students engaged with the program. This evaluation was not conducted using an experimental methodology (participants assigned, guided through the research intervention), nor was the research conducted under ‘ideal conditions’. There was no requirement or expectation that students would participate in a minimum number of sessions (such as in Galante et al., 2018), instead the program was entirely self-guided and self-managed. Students did not receive reminders, alerts or notifications to complete any of the provided materials, unlike other trials of intervention programs (Cavanagh et al., 2013; Hall et al., 2018; Světlák et al., 2021). On one hand, this means we are unable to ascertain if the changes in the outcome measures are likely to be due to the program itself, however on the other hand we can track, record and report what students’ *actual* behaviour is and test its efficacy in a real-world setting.

Whilst over 800 students voluntarily participated in this study, less than half, 37.9% and 40.1%, logged into the LMS program and accessed the Introduction section and Mindfulness activity sections, respectively. This study is the first to our knowledge to capture students’ self-directed engagement with an online university wellbeing program via LMS learning analytics. The pattern of use across the semester is shown in Figure 1.

The majority of the students who logged in to access the materials did so in the first few weeks of the semester (weeks 1–3), with the highest total login recorded in week 2. We hypothesise that this activity represents the students who tried the mindfulness activities (potentially for the first time). Between week 2 and week 3, there was a large drop in student activity, a second but smaller reduction between week 4 and week 7, and thereafter activity remained relatively stable until the second last week of semester (week 11). The decline in engagement between week 4 and week 7 may represent the students who were ‘actively’ participating, but then reduced activity before the middle of semester. We do not currently have nuanced data to explain what led to student disengagement. Potential reasons may be that students were overwhelmed with course materials and this was one of the additional tasks that ‘dropped off’, students who are stressed may not have had the confidence to self-manage their wellbeing via these means, and finally it may explain students’ dissatisfaction with the program.

From week 7 (middle of the semester) to week 11 (the second last week of the teaching semester), login activity remained stable and it appears that the group of students who remained engaged after the lull of mid-semester, continued to practice during the second half of semester. It is hypothesised that the small dip in activity in week 11 could be caused by students deciding to prioritise other activity or tasks in the week prior to the final teaching week. In future research it would be valuable to extend the current study by exploring the level and pattern of engagement between different types of students. For example, comparing engagement between cohorts of students such as by year level (i.e., first year versus third year), as well as by potential discipline of study.

#### RQ3. What Are Students’ Perceptions of Barriers and Motivations Towards Engaging in the Monash Online Mindfulness Program?

According to the Theory of Planned Behaviour (Ajzen, 1991) intention to perform a given behaviour is associated with attitudes, subjective norms, and perceived behavioural control (Armitage and Conner, 2001). The Theory of Planned Behaviour has been applied to and shown effectiveness in behaviour change and health wellbeing interventions (McEachan et al., 2011; Steinmetz et al., 2016). However, Sniehotta et al. (2014) has argued that the Theory of Planned Behaviour lacks validity and the limited predictability of actual behaviours. Sniehotta et al. (2014) proposes that theories not relying on assumptions about cognitions such as theories relating to self-regulation, temporal dynamics, and other approaches that involve integrating multiple theories and approaches may be more beneficial in explaining health behaviour change. Nevertheless, the goal of understanding students’ attitudes in the current study, including their perceived barriers and motivations at the very least, will provide practical implications that are worthwhile considering when implementing a university wellbeing program.

All follow-up participants completed the items rating the perceived barriers and motives, and no statistical differences were found based on engagement level. The most common barrier reported by more than four out of five (84.2%) students was ‘remembering to or creating routine’, closely followed by ‘finding more time in my schedule’, which was reported by 73.2%. Together, this indicates that students perceive that they have limited time, and that wellbeing and self-care activities need to fit into their existing busy. ‘Easily accessing the mindfulness activities on other devices (e.g., phone, tablet) or locations (e.g., on the train)’, was a barrier for 68.9% of the follow-up participants. This demonstrates that only accessing the program via the university’s LMS is a potential barrier for students who want greater flexibility in time, location and convenience.

More than four out of five (86.4%) students reported that ‘seeing that I am benefiting from mindfulness (e.g., general wellbeing and stress)’ would encourage their engagement with a mindfulness program. This was the most significant and common motivator. Perhaps this finding can be likened to spending time on studying or improving an assignment and determining if that time spent was fruitful based on a mark improvement. The next most important motivator was ‘receiving reminders about the mindfulness program (e.g., alters, notifications)’. Sixty-four percent of the sample agreed with this item indicating that most students believe that having further assistance or guidance would encourage them to practice mindfulness. In Hall et al.’s (2018) randomised controlled trial, written reminders were effective in improving mindfulness practice adherence as well as study retention. However, there is also research that suggests students are overwhelmed with information provided through email (Hara, 2000) and discussion forums posts at the start of the semester, and that reminders throughout the semester may be of greater use when they need it most (Lewis et al., 2020). The communication preferences expressed by students versus what has shown to be effective is a balance and demonstrates the complexities in a real-world implementation. Finally, 62.7% of the sample reported that they would be more likely to practice mindfulness ‘if [their] friends and family were also interested in mindfulness’. This supports the notion that engaging in health and wellbeing related activities is impacted by social influences (Umberson et al., 2011) and is consistent with the reported social and cultural barriers reported by Lyzwinski et al. (2018).

These preliminary findings are vital in understanding the motives and processes that students are considering when deciding to engage in wellbeing related activities in general as well as in those that related to self-managed mindfulness programs. To summarise, this study suggests that students are concerned about the perception of lack of time, being able to notice that engagement is ‘working’ and they are seeing benefits, pragmatic considerations that would provide convenience, and finally that the acceptance and practice of mindfulness by others that are close to them also participate in mindfulness.

### Additional Findings

#### Dropout

Participants who dropped out of the study (defined by not completing the follow-up survey) had significantly greater perceived stress levels at baseline than participants who did not dropout (completed the follow-up survey). This might imply that students who are in greater need of stress reducing interventions (because they are more stressed), are also the students who disengage the most. In comparison, students who are ‘less’ stressed are engaging with the materials more (i.e., choosing to finish their research participation). However this explanation is simplistic and we do not currently have the data to fully explain this phenomenon. It is hypothesised that the students that dropped out are not homogenous; rather this group may be made of up students who have (1) continued to engage with the intervention program but did not complete the follow-up survey, (2) disengaged with the intervention because they were too distressed, or (3) disengaged with the intervention for another reason other than their wellbeing state (e.g., did not like the intervention). Future research would benefits from understanding how much of the ‘study dropout rate’ is actual program dropout or just study/follow-up dropout, and reasons for disengagement should be separated from ‘study dropout’.

#### COVID-19 Implications

It was outside the scope of this study to deeply explore the impact of COVID-19 on students’ experiences and engagement with the mindfulness-program. However the impacts of COVID-19 as reported by the students at follow-up are consistent with the findings of Browning et al. (2021), a large survey of over 14,000 college students in the United States in 2020. The most commonly reported impacts in the current study and Browning et al. (2021) include isolation, and feelings of reduced wellbeing and increased stress/anxiety. Additionally, a large proportion of students in the current sample (52.4%) expressed additional financial burden compared to only 4.2% of the sample expressing financial worry in Browning et al. (2021).

### Limitations

The main aim of this study was not to attribute change in student wellbeing, stress and mindfulness directly to the mindfulness program, and hence an experimental, randomised, and controlled design was not used. Therefore, an inherent limitation is that we cannot determine if the changes reported in the outcome measures are in fact due to participation in the mindfulness program. Secondly, a technological limitation with the LMS functionality for recording log data impacted on the user experience. Initially it was intended that students would be able to tick off completed activities, and this history would be available at the next login. This feature provides a sense of accomplishment, and allows participants to self-monitor their progress in health-related online interventions and therapies (Mehra et al., 2020; Hanley and Wyatt, 2021). Additionally, this would provide more specific and detailed LMS log data. Unfortunately, due to limitations with the LMS, this was not a feature of the current program. Thirdly, only the duration of the students’ LMS log in was recorded. This data represents the length of time that an individual had the program webpage open. It does not track interactions with the webpage such as scrolling, and starting, pausing or finishing an audio file. Therefore, whilst this study provided valuable information on students’ login patterns, this limitation must be considered.

An inherent limitation in research in this context is that (a) students self-selected into this study and are therefore more likely to be interested in exploring their wellbeing (compared to the wider student population), and (b) the high follow-up survey dropout rate (71.7%) does not necessarily represent an accurate picture of the *whole* samples’ change in wellbeing, stress and mindfulness. As mentioned earlier, study dropout includes both students who did *and* did not continue to engage with the program materials. Whilst on one hand the large *study* dropout rate is a limitation in our potentially biased representation of the study findings, it should not be disregarded it may be indicative of *actual* engagement with the program and assists us in understanding students’ behaviours.

Finally, an important limitation of the mindfulness program was the lack of follow-up with students at the end of the research study, as well as the lack of additional support provided to students who needed greater mental health support such as 1:1 assistance, or who experienced adverse events.

### Strengths

The greatest strength of this study was the vision, study design and methodology which allowed us to assess the ecological validity of an online wellbeing program for university students. This has advanced existing understanding and tested current assumptions on how students actually engage with wellbeing programs in university settings. Secondly, this study is one of only a few to examine a fully online, large scale, university-wide, implementation of a wellbeing and mindfulness program. Whilst there is significant value in determining the effectiveness of mindfulness interventions in controlled settings, it is difficult to draw conclusions on their impact as an actual university resource. The concluding remarks of controlled studies often recommend that interventions be included as part of orientation or course programs, but they lack the specific guidance and discussion on how such programs can be implemented successfully. This study provides a valuable first step in moving beyond trials and into a real understanding of how to best implement mindfulness successfully into university settings.

Finally, by collecting LMS log data, we were able to report students’ actual behaviour and engagement with the online program. This gave into when students engage with wellbeing resources when they are fully self-managed with minimal prompting. For example, the decline in engagement found after week 3 and 11 (of a 12-week semester) is useful in helping universities to understand when reminders should be targeted. Whilst LMS data has been used for tracking and monitoring student progress (You, 2016), it is largely missing from university wellbeing programs evaluations, and most studies rely heavily on self-report data.

### Implications and Future Research

The three main themes and implications of this research and thus leading to suggestions for future research include: (1) students who did not engage with the program at all and students who dropped out of the study appear to be at risk of greater stress; (2) there is a complex and non-linear relationship between program engagement and wellbeing, perceived stress and mindfulness benefits; and (3) the barriers and motivations as expressed by students can help us to potentially improve the program for future implementations. Future research could extend the project evaluation into the end of semester exam period, however this may also have implications on follow-up survey completion and finally future studies could consider examining participants’ perceived barriers and motivations in the baseline survey. Finally, we believe that future research could greatly benefit from drawing upon the implementation science literature base.

Firstly, students who are the most stressed are the students who chose not to engage with the program, disengaged and dropped out of the research study and thus we were unable to measure their end of semester mental wellbeing levels. This implies that the students who are most ‘at need’ are not being reached, and are more likely to disengage. It would be very worthwhile to understand the challenges these students are facing and whether or not this disengagement also extends to academic related activities. A more nuanced understanding of dropout is necessary. Specifically, how much of the ‘study dropout rate’ is actual program dropout or just study/follow-up dropout, are reasons for disengagement associated with dissatisfaction with the program, and reasons for disengagement should be separated from ‘study dropout’.

The second major implication of this real-world university implementation is understanding that online mindfulness program engagement does not necessarily have a linear relationship with improvement in wellbeing, stress and mindfulness. As confounding factors were not controlled for, this study demonstrates the complexity in the real-world efficacy when conditions are not ‘ideal’. Furthermore, the *size* or *amount* of improvement seen in the outcome measures was also not dependent on *amount* of engagement or interaction with the online mindfulness program. Future research that attempts to explore and explain these non-linear relationships would be very beneficial.

Lastly, this study has provided insight into the perceived barriers and motivating factors that students consider when engaging in a university self-managed online mindfulness-based program. Whilst the findings can be used to inform theoretical implications such as the application of the Theory of Planned Behaviour (Ajzen, 1991), or combined approaches and theories, the findings also provide important practical implications. University students remain concerned about the perceived lack of time, are driven by seeing ‘results’, and make practical and pragmatic considerations (e.g., reminders and alerts, access via other devices such as phone apps or tablets). Finally, students also consider the behaviours of individuals in their social networks.

The core aim of implementation science is to understand how relevant and contextual processes impact on the quality and effectiveness of interventions in real, applied contexts (Kelly, 2012). The implementation science literature base posits that problems often arise when implementing ‘evidence-based’ programs (e.g., randomised controlled trials) in real-world contexts, which do not replicate experimental conditions. The root of these issues is due to the fact that scientific literature and methods often fail to anticipate factors and processes that underlie variability and unpredictability in relation to effectiveness (Kelly, 2012). Kelly (2012) explains that implementation science frameworks influence how interventions are “conceived, designed, and resourced”, and furthermore, provide “preparation, execution, evaluation and sustainability of interventions” in a range of contexts, including in education (p. 6). As such, we recommend that future research should extend beyond pure evaluation in experimental conditions and draw upon the implementation science literature base and consider relevant frameworks.

## Conclusion

The current ecological validity and real-world research study has provided a meaningful contribution to the understanding of how students *actually* engage with university online wellbeing and mindfulness programs. The total sample included 833 participants with learning analytics collected from LMS log data revealing that 41.2% of the total sample accessed the online mindfulness program at least once over the 12-week semester during COVID-19 in 2020. Whilst improved wellbeing, perceived stress and mindfulness was seen in participants who *Actively engaged* with the program (3 or more weeks of logins), the relationship between engagement and efficacy in outcome measures is non-linear and further investigation is needed to understand this complex relationship. This study has highlighted the complexities of implementing a real-world, large, university-wide, online mindfulness-based program that was accessible fully online via the tertiary institutions’ LMS student orientation site. It has shown that when ideal experimental conditions are not provided and students fully self-manage their own wellbeing, a deeper and more nuanced understanding of student behaviours is necessary. This research is the first step in this process and we encourage institutions that are not yet actively supporting student wellbeing to do so, to evaluate the implementation of these programs or resources, and finally to share these findings by contributing to empirical research on university student wellbeing initiatives.

## Data Availability Statement

The datasets generated for this study are available on request to the corresponding author.

## Ethics Statement

The studies involving human participants were reviewed and approved by Monash University Human Research Ethics Committee. The patients/participants provided their written informed consent to participate in this study.

## Author Contributions

JC collected the data, conducted data analysis and interpretation, and prepared the first draft of the manuscript. MM and SM provided expert guidance and data interpretation. All authors conceptualised and designed the study protocol. All authors contributed to the revision of the manuscript and approved the submitted version.

## Conflict of Interest

The authors declare that the research was conducted in the absence of any commercial or financial relationships that could be construed as a potential conflict of interest.

## Publisher’s Note

All claims expressed in this article are solely those of the authors and do not necessarily represent those of their affiliated organizations, or those of the publisher, the editors and the reviewers. Any product that may be evaluated in this article, or claim that may be made by its manufacturer, is not guaranteed or endorsed by the publisher.
