# Supplementary material for: A Self-Managed Online Mindfulness Program in a University-Wide Learning Management System Orientation Site: A Real-World Ecological Validation Study
Source: Front Psychol. 2022 May 6;13:869765. doi: 10.3389/fpsyg.2022.869765 (PMC9121920; doi:10.3389/fpsyg.2022.869765)
Supplement: Supplementary file 1 [file Table_1.DOCX]

*Note. Data are Mean Rank. WEMWBS = Warwick Edinburgh Wellbeing Scale. PSS = Perceived Stress Scale. FFMQ = Five Facet Mindfulness Questionnaire.*

Supplementary Material 1

*Kruskal-Wallis between engagement type groups for change in wellbeing, stress and mindfulness*

|  | | | | Kruskal-Wallis | | | |
| --- | --- | --- | --- | --- | --- | --- | --- |
|  | No engagement | Trial engagement | Active engagement | N | *df* | *H (corrected for ties)* | *p* |
| Change WEMWBS | 112.98 | 120.92 | 125.25 | 236 | 2 | 1.330 | 0.514 |
| Change PSS | 127.00 | 105.07 | 109.07 | 230 | 2 | 5.332 | 0.070 |
| Change FFMQ | 110.93 | 108.88 | 129.90 | 229 | 2 | 3.889 | 0.143 |

*Note. Drop out = participants that did not complete the follow up survey. Did not drop out = participants that completed baseline and follow-up surveys. WEMWBS = Warwick Edinburgh Wellbeing Scale. PSS = Perceived Stress Scale. FFMQ = Five Facet Mindfulness Questionnaire. CI = confidence interval. Bold = significant p < .05.*

|  |  | |  |  | |  | Independent samples *t*-test | | | | | | |
| --- | --- | --- | --- | --- | --- | --- | --- | --- | --- | --- | --- | --- | --- |
|  | Drop out | |  | Did not drop out | |  |  | | | | | 95% CI | |
|  | *n* | *M (SD)* |  | *n* | *M (SD)* |  | *t* | *df* | *p* | Mean difference | *Std err diff.* | Lower | Upper |
| WEMWBS | 597 | 45.32 (8.19) |  | 236 | 45.50 (8.58) |  | -0.3 | 831 | 0.779 | -0.18 | 0.64 | -1.43 | 1.07 |
| PSS | 603 | 21.07 (5.85) |  | 230 | 19.81 (5.63) |  | 2.8 | 831 | **0.005** | 1.26 | 0.45 | 0.37 | 2.14 |
| FFMQ | 604 | 54.20 (8.63) |  | 229 | 55.12 (8.88) |  | -1.4 | 831 | 0.175 | -0.92 | 0.67 | -2.24 | 0.41 |

Supplementary Material 2

*Participant drop-out analyses on outcome measures at baseline*
